# Q‑Band Double Quantum Coherence ESR for Sensitive Nitroxide-Based Distance Measurements

**DOI:** 10.1021/acs.jpcb.5c03169

**Published:** 2025-07-07

**Authors:** Alysia Mandato, Nicholas A. Moriglioni, Sunil Saxena

**Affiliations:** Department of Chemistry, 6614 University of Pittsburgh, Pittsburgh, Pennsylvania 15260, United States

## Abstract

Recent advances in pulsed dipolar spectroscopy have driven the sensitivity of the technique to physiologically relevant concentrations and extended its applicability to more complex biological systems. In this work, we establish double quantum coherence (DQC) ESR at Q-band as a practical technique for distance measurements in doubly nitroxide-labeled proteins. We show that 8 ns π pulses provide efficient excitation of the double quantum transition at Q-band, though more accessible 12 and 16 ns pulses also yield strong signals. Additionally, we show that the field dependence of the DQC signal is sensitive to the relative orientation of the two g-tensor axes. With careful spectrometer tuning, signal-to-noise ratios greater than 100 were possible in just 11 min of acquisition for a dipolar evolution time of 1.5 μs with 50 μM protein samples. We also show the effects of varying levels of deuteration on the sensitivity of the DQC signal, which can further extend dipolar evolution times and the measurable distance range. The DQC data is in good agreement with theoretical expectations and displays negligible contributions of intermolecular interactions on the DQC background signal at protein concentrations up to 100 μM. Overall, Q-band DQC offers distinct advantages for dual nitroxide-labeled proteins, including deep dipolar modulations, minimal background decay at micromolar concentrations, and increased sensitivity with the incorporation of deuterium. These findings position Q-band nitroxide DQC as a broadly accessible technique for pulsed ESR distance measurements.

## Graphical abstract

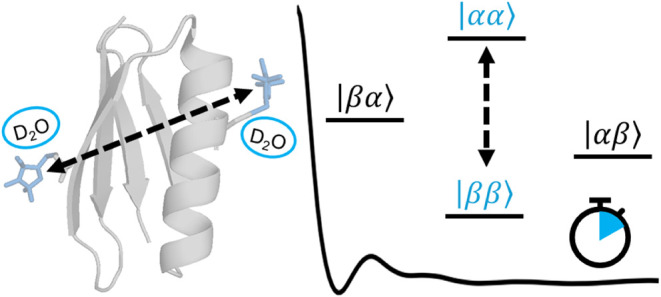

## Introduction

Enhancement of signal sensitivity is essential to extend the reach of biophysical techniques. Highly sensitive measurements are particularly beneficial when working with biomolecules that are difficult to express in large quantities or prone to aggregation at high concentrations. Moreover, many biologically relevant proteins, including transcription factors and transport proteins, exist at nanomolar levels in cellular environments.^1–3^ Detecting such low concentrations with high sensitivity is crucial for measuring physiologically significant properties, such as submicromolar binding affinities and equilibrium dynamics, *in vitro*.^4,5^ In addition to measurements at low concentrations, high signal sensitivity is key to uncovering accurate structure–function relationships within cells.^6–10^ As a result, many biophysical methods are being pushed to their sensitivity limits to meet these demands.

Among the biophysical techniques, electron spin resonance (ESR) spectroscopy stands out as a highly incisive method for measuring biomolecular structure and dynamics. ESR spectroscopy is selective only to unpaired electrons, which are typically site-specifically introduced to otherwise diamagnetic biomolecules via spin labels.^11,12^ Once spin-labeled, pulsed dipolar spectroscopy (PDS) can be exploited to determine the distance distribution between two spin labels.^13–18^ In PDS techniques, biomolecular distance constraints ranging from 1.5 to 16 nm are obtained from distance-dependent magnetic dipolar couplings between electron spins.^13,14,19,20^ Such long-range distance constraints are incisive probes of conformational changes,^21–25^ relative arrangements of biomolecules,^26,27^ and measurement of biomolecular interactions and assembly.^28–31^

Several PDS methods have been developed to measure distance constraints. Conceptually these methods involve either pulses that irradiate at a single-frequency^15–18,32–35^ or at two different frequencies.^13,14,36^ Of these PDS techniques, the four-pulse Double Electron Electron Resonance (DEER or PELDOR) experiment is the most widely used especially due to the modest requirements on the lengths of pulses.^37^ Consequently much attention has also focused on understanding the optimal ways of performing the DEER experiment^37–39^ as well as on the analysis of the data.^40–45^ The double-frequency nature of DEER requires careful placement of the observer and pump frequencies to optimize spectral excitation while also avoiding pulse overlap. Nitroxide spin-labeled proteins are well suited for DEER experiments, as the typical pulses used can be applied at two different frequencies to cover a majority of the spectrum without overlap. In addition, significant gains in sensitivity have resulted from the advent of Q-band DEER^46^ and shaped pulses to efficiently excite the spectrum.^47–49^ Nevertheless, a limitation and loss of resolution in DEER is the presence of contributions from intermolecular dipolar interactions.^37,50^

Single-frequency PDS techniques draw on other mechanisms to measure the dipolar interaction. For example, Relaxation Induced Dipolar Modulation Enhancement (RIDME) spectroscopy relies on the stochastic flipping of a coupled spin due to longitudinal relaxation rather than an inversion pulse. Since the inversion of the dipolar interaction is independent of pulse bandwidths and frequency offsets,^51,52^ RIDME can be applied to spin labels with broad EPR spectra. Additionally, the nature of the RIDME experiment makes it especially useful for studying orthogonal spin pairs rather than identical pairs of spins.^16,53^ In particular, the combination of Cu­(II) and nitroxide is especially advantageous because of the efficient observation of the nitroxide spin.^4,51,54–56^ Furthermore, the fast relaxation of the metal ion spin leads to a greater number of spin-flips during the course of the experiment, which enhance the modulation depth.^57–59^ These advantages, however, come at the expense of a more complicated background signal.^52,60,61^

In contrast, Double Quantum Coherence (DQC) spectroscopy is a single-frequency method that can be used on identical pairs of nitroxide spins. In this experiment, an echo that follows the double quantum coherence pathway is measured. Since this coherence is due to the dipolar interaction, the signal is sensitive to the distance between spins. Nevertheless, the experiment necessitates short and intense pulses for efficient single to double quantum coherence transfers.^33,62,63^ This requirement has largely limited the use of DQC to spins exhibiting a narrow spectrum, like trityl-based radicals,^9,53,64–67^ or at lower microwave frequencies (X- or Ku-band) for nitroxides and copper.^26,34,68–75^

Recent efforts have focused on calibrating the sensitivity of PDS techniques to enable measurements at low protein concentrations. While DEER is the most widely used PDS method, its reliability in distance measurements diminishes below 100 nM due to relatively shallow modulation depths and low signal-to-noise ratios after hours of collection.^76,77^ On the other hand, RIDME has demonstrated distance measurements at concentrations in tens of nanomolar concentrations,^5,54^ and remarkably, as low as 10 nM.^78^ These measurements rely on orthogonally spin-labeled proteins whereas the vast majority of PDS studies use nitroxide-labeled systems. To advance PDS sensitivity in a way that is both practical and broadly applicable, there is a need for a technique that combines high sensitivity with commonly used double nitroxide spin labeling.

Nitroxide DQC is typically performed at X- and Ku-band due to the availability of hardware that can create narrow pulses at these frequencies. Unlocking the gain in sensitivity by operation at Q-band was considered challenging for DQC given the difficulty of achieving short excitation pulses. However, with recent advancements in technology, we recently demonstrated the first application of DQC at Q-band using nitroxide-based spin labels. This work shows that at Q-band, nitroxide-based DQC stands out with its deep modulation depths and low background signals, which both enhance sensitivity as well as reduce analysis uncertainties.

In this work, we outline the use of Q-band DQC by evaluating the effects on changes to pulse lengths and separations of the six-pulse DQC sequence. More importantly, we explore gains in sensitivity of DQC measurements by measuring the impacts of deuteration, modulation depth, and background function. This systematic examination of the Q-band nitroxide DQC experiment will promote the broader use of the method as a sensitive tool for distance measurements between identical spin pairs.

## Experimental Methods

### Protein Preparation and Spin Labeling

Expression and purification of the E15C/K28C mutant of the B1 immunoglobulin binding domain of protein G (GB1) were performed as described previously.^79,80^ Following purification, the GB1 mutant was reacted with 5 mM dithiothreitol (DTT) in a total volume of 1 mL for 2 h at room temperature to reduce any disulfide formation. The reducing agent was removed using five 5 mL HiTrap desalting columns (Cytiva Life Sciences) and 1× PBS pH 7.75. The protein fraction was collected into a solution of 20-fold molar excess of (1-oxyl-2,2,5,5-tetramethylpyrroline-3-methyl)­methanethiosulfonate spin label (MTSSL, Sigma-Aldrich) per cysteine. The labeling reaction was incubated overnight at 4 °C. Excess spin label was removed by collecting the first elution peak from the HiTrap desalting columns using 1× PBS pH 7.75. The protein concentration was quantified using a NanoDrop 2000 spectrophotometer (Thermo Scientific) (GB1 ε_280_ = 9970 L mol^–1^ cm^–1^). The labeling efficiency was determined by continuous-wave ESR using the quantitative ESR spin-counting experiment on XEPR (Bruker BioSpin). Spin-labeled protein solutions were stored in 100 μL aliquots in PBS pH 7.75 at −80 °C.

The protocol for protein expression for the deuterated GB1 mutants was adopted from a published uniform isotope labeling method.^81^ The expression medium consisted of 1.5× M9 minimal salts (72 mM Na_2_HPO_4_, 33 mM KH_2_PO_4_, 13.5 mM NaCl) in 980 mL of sterilized deionized water, pH 7.4, and 20 mL of “Master Mix #1” (2 g glucose, 1 g NH_4_Cl, 2 mM MgSO_4_, 100 μM CaCl_2_, 200 mg yeast extract, and 100 mg ampicillin in sterilized deionized water). Nondeuterated glucose was used in Master Mix #1 because it was depleted prior to protein expression. The 1 L solution was allowed to equilibrate at 37 °C for 10 min. Next, 15 mg each of FeSO_4_ and ZnCl_2_ were added to the expression medium and allowed to equilibrate for 10 min at 37 °C. After equilibration, 26 mL of overnight growth culture grown in LB media were added and grew at 37 °C with mixing at 250 rpm. The optical density was monitored at 600 nm (OD_600_) until it reached ∼ 0.8. Next, 3 g of 97–99% ^2^H ISOGRO algal amino acid mixture (Sigma-Aldrich) was dissolved in 50 mL of D_2_O containing 1 g glucose, 2 mM MgSO_4_, and 100 μM CaCl_2_. This mixture was directly added to the expression medium, and it was mixed at 25 °C for 10 min to equilibrate. Protein expression was induced with 500 μL of 1 M IPTG and maintained at 18 °C with shaking at 250 rpm overnight. Protein purification with anion exchange and size exclusion chromatography was performed in protic solvent and buffers, but final storage conditions were in 1× PBS pH 7.5 in D_2_O.

### ESR Spectroscopy

Continuous-wave (CW) ESR experiments were performed on the Bruker ElexSys E680 FT/CW spectrometer with a Bruker ER4122 SHQE-W1 high-resolution resonator at X-band (∼9.8 GHz) frequencies. For the room temperature measurements, 20 μL of 50 μM protein were drawn into a Pyrex capillary tube (0.8 mm I.D. × 1 mm O.D.), sealed, and placed into ESR sample tubes (3 mm I.D. × 4 mm O.D.). CW-ESR data were acquired at a center-field of 3512 G with a sweep width of 150 G and for a total of 1024 data points using a conversion time of 20.48 ms. A modulation frequency of 100 kHz was used with a modulation amplitude of 1 G.

Pulsed ESR experiments were performed at 50 K at Q-band on an upgraded Bruker ElexSys E580 FT/CW spectrometer with a Bridge12 Technologies QLP Q-band resonator, a Bruker SpinJet AWG, and a 300 W TWT amplifier. Samples were prepared in clear fused quartz capillary tubes (1.1 mm I.D. × 1.6 mm O.D.) and were flash-frozen in liquid methylacetylene-propadiene propane (MAPP) gas with 20% (v/v) glycerol or d_8_ glycerol as a cryoprotectant.^82^ The total sample volume was 10 μL, and the protein concentration was 50 μM, unless otherwise noted. Double quantum coherence (DQC) experiments were performed using the six pulse sequence (π/2)–t_p_–π–t_p_–(π/2)–t_d_–π–t_d_–(π/2)–(t_m_–t_p_)−π–(t_m_–t_p_)–echo.^18^ A 256-step phase cycling program was used (64 steps combined with CYCLOPS).^62^ All measurements were obtained with 20 shots per point and a 1 ms shot repetition time. Other DQC parameters are discussed in the Results section.

## Results and Discussion

In this work, we expressed and purified a 15C/28C mutant of the B1 immunoglobulin binding domain of protein G (GB1)^83–85^ using previously established protocols.^79,80^ The cysteine residues were labeled with (1-oxyl-2,2,5,5-tetramethylpyrroline-3-methyl)­methanethiosulfonate spin label (MTSSL) as described in Experimental Methods. GB1 is an established model protein that is used extensively in ESR.^10,86–88^ Continuous-wave ESR was performed to estimate labeling efficiency of the 15R1/28R1 GB1 (Figure S1 and Table S1). Pulsed ESR experiments were performed using the Bridge12 Q-band loop gap resonator with a 10 μL sample of 50 μM 15R1/28R1 GB1 in nondeuterated buffer.

### Microwave Frequency and Magnetic Field Optimization

We first measured the bandwidth of the Bridge12 QLP Q-band resonator to find the optimal spectrometer frequency for the subsequent DQC experiments. We performed a bandwidth nutation experiment to measure the pulse as a function of operating frequency. Figure 1A shows the optimal π pulse length versus operating frequency in open gray circles. Figure 1B shows the pulse sequence of the nutation experiment.^89,90^ Briefly, we incremented the length of the first pulse and monitored the time evolution of the resulting magnetization using a Hahn echo sequence (π/2 – τ – π – τ). As a result, a π pulse of 8 ns was consistently achieved over a 200 MHz bandwidth, ranging from 34.4 to 34.6 GHz. Because the sensitivity of the DQC experiment benefits greatly from short pulses to effectively excite the double quantum coherence, we focused our experiments on the region where 8 ns π pulses could be applied.

**Figure 1:**
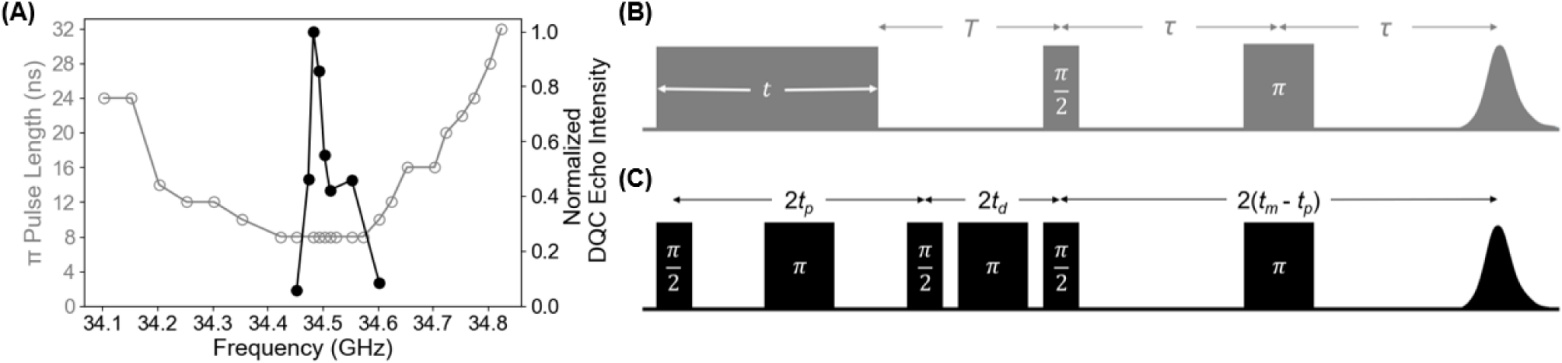
(A) Nutation bandwidth of the resonator based on optimal π pulse length (open gray circles) and the normalized DQC echo area as a function of operating frequency (black circles). (B) The echo-detected transient nutation pulse sequence. A delay time of *T* is inserted after the first pulse of variable duration *t*. The change in magnetization caused by the first pulse is detected by the Hahn echo generated by (π/2) – τ – (π) – τ. (C) The 6-pulse double quantum coherence (DQC) sequence. The DQC is generated by the pulse “sandwich” (π/2) – *t*_*d*_ – (π) – *t*_*d*_ – (π/2). The last π pulse refocuses the coherence to produce the echo at time 2*t*_*m*_ + 2*t*_*d*_. The DQC signal is recorded as *t*_ξ_ = *t*_*m*_ – 2*t*_*p*_.

To further explore the effect of spectrometer frequency, we measured the intensity of the DQC echo at various frequencies using 4 ns π/2 and 8 ns π pulse lengths. Each data point, shown in Figure 1A as black circles, was collected at the maximum of the field-swept electron-spin echo (FS-ESE) spectrum of GB1. The DQC echoes were generated using the pulse sequence shown in Figure 1C. In this experiment, we set the first and last pulse separations, t_p_ and (t_m_ – t_p_), equal to each other, which made t_m_ = 0. This setup allowed us to collect the echo at the maximum intensity of the DQC time trace, corresponding to the echo with the best signal-to-noise ratio. We collected echoes in 10 MHz intervals within the frequency range where the π pulse is 8 ns (34.45–34.6 GHz). For this sample, we observed the highest DQC echo intensity at 34.48 GHz. The data highlights the sensitivity of the measurement to spectrometer frequency and the importance of determining the optimal spectrometer frequency to achieve the most intense DQC echo. The variation in intensity across the frequency range may be due to B_1_ field inhomogeneity and the consequential lack of adiabaticity.^91–93^

Figure 2A shows the FS-ESE spectrum of 15R1/28R1 GB1 collected at 34.48 GHz (cf. black solid line). We also plotted the inversion profile of an ideal 8 ns rectangular pulse, simulated in EasySpin,^94^ as the gray shaded function. Next, we collected the DQC echo as a function of the external magnetic field B_0_. Figure 2B shows the integrated DQC signal as gray circles overlaid on the FS-ESE spectrum (details of integration provided in Figure S2). Note that both data sets were normalized. The data shows that the DQC echo reaches its largest area at the maximum of the FS-ESE spectrum. Interestingly, the intensity of the DQC echo does not directly follow the intensity of FS-ESE spectrum, and the DQC echo intensity is substantially lower at higher fields.

**Figure 2:**
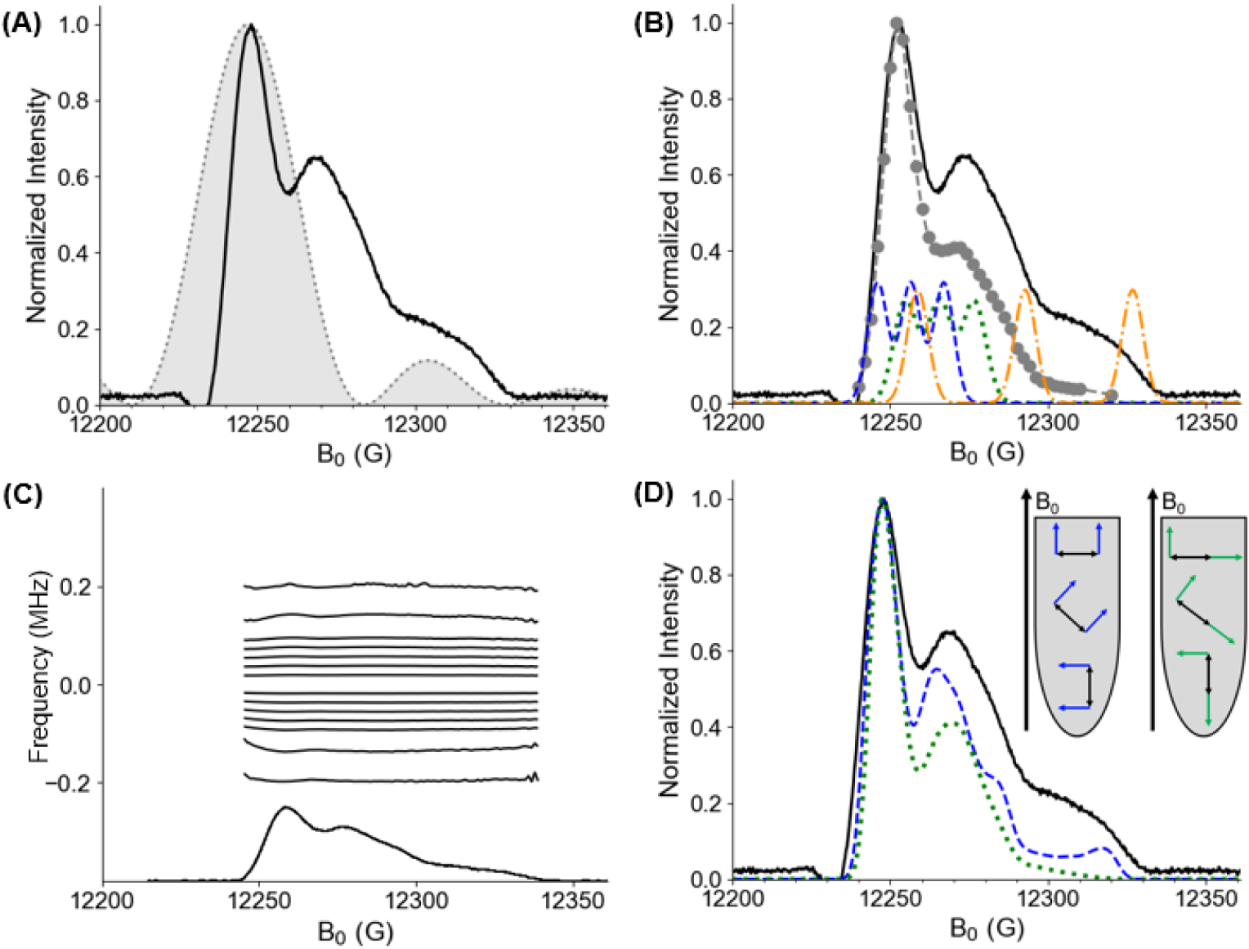
(A) Q-band field-swept electron-spin echo (FS-ESE) spectrum of 15R1/28R1 GB1 (black solid line). The normalized excitation bandwidth of an 8 ns rectangular pulse (gray shaded area) is overlaid on the spectrum. (B) The DQC echo area as a function of applied magnetic field position (gray circles with dashed line). The FS-ESE spectrum is shown in black for comparison. Overlaid on the spectrum are the line shapes for a spin with the g_*xx*_ (blue dashed), g_*yy*_ (green dotted), and g_*zz*_ (orange dash-dotted) aligned with the applied magnetic field. (C) Normalized contours in the FS-ESE spectrum, indicating constant phase memory times (T_M_) as a function of magnetic field. (D) The DQC intensity was calculated for two cases, shown in the inset. The relative orientations for the two g-tensors are described by five Euler angles, which are defined in the Supporting Information. In the two cases, these angles were set to (0°, 0°, 0°, 0°, 0°) and (90°, 90°, 90°, 0°, 0°) for α, β, γ, ξ, and φ, respectively. The angles are defined in Figure S3. The standard deviation was 25° for each angle. The probability of mutual spin excitation within a spin pair as a function of magnetic field is plotted for the two cases. A FS-ESE spectrum is overlaid in black for comparison.

We carried out a 2D FS-ESE experiment to examine whether variations in phase memory time (T_M_) across the spectrum^95–97^ are responsible for the changes in the DQC signal with field. Figure 2C shows the normalized contour plot for the 15R1/28R1 GB1 sample at 50 K. In this experiment, the Hahn echo as a function of magnetic field was collected (direct dimension) at various values of the separation between the two pulses (indirect dimension). The data was Fourier transformed in the indirect dimension and normalized as described previously.^95,96^ We find that the T_M_ is constant as a function of B_0_, ruling out the role of T_M_ for the lowered relative intensity of the DQC echo at higher fields when compared to the FS-ESE spectrum.

We therefore attribute the differences between the FS-ESE data and DQC data (cf. Figure 2B) as due to the variation in the excitation of the DQC coherence with magnetic field. Figure 2B shows the simulated line shape for a spin with the g_*xx*_, g_*yy*_, and g_*zz*_ aligned with the applied magnetic field, B_0_. It is evident that there exists much overlap in the spectral frequencies around the maximum of the FS-ESE spectrum (i.e., at ca. 12250 G). The pumping of the double quantum transition is optimal when both spins in the pair have similar resonance frequencies.^33,63^ Thus, the DQC signal is expected to be optimal at this field position, irrespective of the relative orientations between the g-tensors of the two nitroxides. On the other hand, pumping of the DQC coherence at higher fields is suboptimal because of the reduced likelihood of both spins being excited.

To further illustrate this effect, we adapted a recently developed computational strategy^98–100^ wherein the signal from pulsed dipolar spectroscopy is calculated by creating an ensemble of spin pairs. In this method, the relative orientations of the g-tensors of each spin in the pair is prescribed, and based on the pulse properties, each spin pair is interrogated to examine the contribution to the signal. The relative orientations of the two g-tensors in the protein are given by five Euler angles.^33^ We examined two cases, as shown in Figure 2D, with the two g_*zz*_ axes oriented “parallel” (blue dashed) and “perpendicular” (green dotted) to each other. The simulated DQC intensity as a function of magnetic field is overlaid onto a FS-ESE spectrum. The intensity of the DQC signal at higher magnetic fields is larger for the parallel orientation compared to the perpendicular because the resonance frequencies of both spins in this g_*zz*_ region are similar. These simulations suggest that the field dependence of the DQC signal can be exploited to measure relative orientations. Such effects have been observed in different contexts.^62,101^

### Sensitivity to Duration of π Pulse

To evaluate the effect of pulse lengths, we collected the DQC echo at 34.48 GHz using a range of π pulse lengths. The data is shown in Figure 3A. At the lowest microwave power, we generated a DQC echo with a 16 ns π pulse with 3 dB attenuation. When we reduced the attenuation to 2 dB, we observed a 6% increase in echo area with a 12 ns π pulse, as depicted in Figure 3B. Eliminating all microwave attenuation produced in an 8 ns π pulse, which resulted in the largest DQC echo with an area 9% greater than that of the 16 ns pulse. Reducing the pulse length further to 4 ns dramatically reduced the size of the echo, since a π pulse could not be realized. These data highlight the importance of strong pulses in nitroxide-based DQC, and the results are consistent with previous work at X- and Ku- bands.^72,73,102,103^ Nevertheless the data in Figure 3B is encouraging. In our work we were able to achieve 8 ns π pulses by a combination of a 300 W TWTA and the Bridge12 resonator. Figure 3B suggests that DQC on nitroxides at Q-band should be efficient even with more widely available instrumentation that can typically achieve 16 ns π pulses.

**Figure 3:**
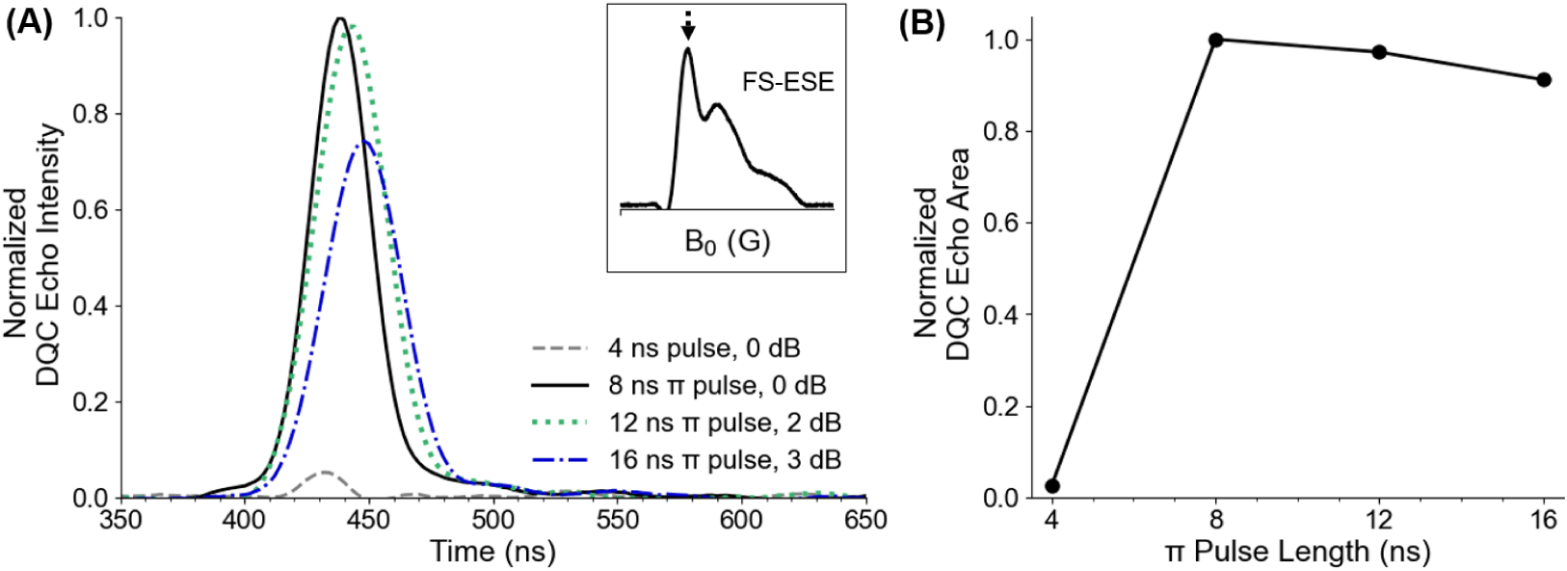
(A) Normalized DQC echo obtained with 4 (gray dashed), 8 (black solid), 12 (green dotted), and 16 (blue dash-dotted) ns π pulse lengths at 0, 0, 2, and 3 dB microwave power attenuations, respectively. The 4 ns pulse is not a true π pulse. The echoes were collected at the maximum of the FS-ESE spectrum, as shown by the inset. (B) Normalized DQC echo area as a function of π pulse length.

### Impact of Deuteration on DQC Sensitivity

Next, we determined the impact of deuteration to the sensitivity of the DQC experiment, given that previous findings have demonstrated a significant increase in phase memory relaxation time (T_M_) by the reduction of the electron–nuclear dipolar interaction.^20,104,105^ For comparative purposes, we prepared a 50 μM 15R1/28R1 sample in deuterated buffer and glycerol-d_8_. Additionally, another sample consisted of deuterated GB1 using a cell expression protocol described in the Experimental Methods.^81^ We used liquid chromatography–mass spectrometry to estimate that the deuterated GB1 was at least 70% isotopically labeled (Figure S4).

We first examined the effects of electron–nuclear interactions on the intensity of the DQC echo. For each sample, we collected the DQC echo area as a function of t_d_, the pulse separation between the pulses in the DQC sandwich sequence that generate the double quantum coherence (e.g., the separation between the third and fourth, and fourth and fifth pulses). Figure 4A shows the normalized DQC echo areas collected for a dipolar evolution time of 1.2 μs at the maximum of the FS-ESE spectrum. The normalized DQC echo areas were obtained by dividing by the standard deviation of the noise (σ_n_) in the echoes. As shown in Figure 4A, there is a clear modulation of the echo area as the value of t_d_ is increased in the deuterated samples due to electron–nuclear interactions. The signals exhibit a frequency corresponding to deuterium ESEEM at the Q-band frequency (7.8 MHz).^104,106,107^ In comparison, the echo area for the nondeuterated sample displays only a weak reduction over the range of measured t_d._ Based on these observations, for deuterated samples it is crucial to choose a t_d_ at a maximum of these modulations to achieve the highest sensitivity in DQC experiments. While many reported DQC experiments use short t_d_ values (usually ranging from 20 to 100 ns),^18,63,70,108^ these results demonstrate that optimizing t_d_ for deuterated samples can significantly enhance the DQC echo area and improve overall sensitivity. Additionally, we show that the double quantum coherence decay is not fast, and the DQC echo area does not decay significantly as t_d_ separation is increased, even to values of several hundred nanoseconds.

**Figure 4:**
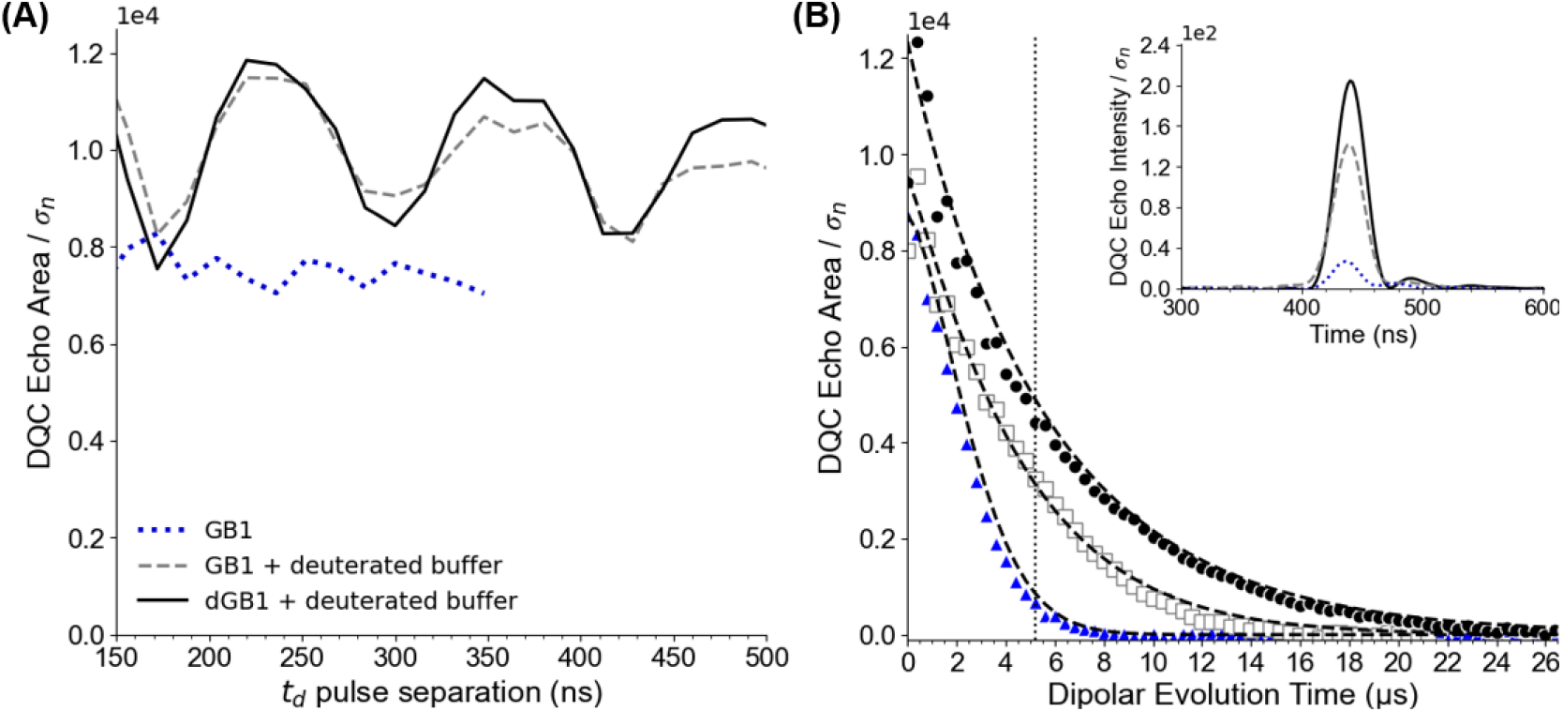
(A) DQC echo area as a function of *t*_*d*_ pulse separation for nondeuterated GB1 (dotted blue), GB1 in deuterated buffer and glycerol (dashed gray), and deuterated GB1 in deuterated buffer (black). The *t*_*d*_ pulse separation was incremented by 16 ns for each data point. (B) The DQC echo area plotted as a function of dipolar evolution time for nondeuterated GB1 (blue triangles), GB1 in deuterated buffer and glycerol (open gray squares), and deuterated GB1 (black circles). The data were collected by incrementing 2*t*_*p*_ and 2­(*t*_*m*_ – *t*_*p*_) by 200 ns each per point. The dashed black lines are the fits of the data by accounting for phase memory time and instantaneous diffusion. The dotted vertical line highlights the example echoes shown in the inset. The inset shows DQC echoes for a dipolar evolution time of 5.2 μs for nondeuterated (dotted blue), deuterated buffer (dashed gray), and deuterated protein (black) solutions.

To further explore the benefits of deuteration, we measured the integrated DQC echo as a function of dipolar evolution time (t_ξ_) for each sample. We sequentially increased the 2t_p_ and 2­(t_m_ – t_p_) pulse separations in the DQC pulse sequence (Figure 1C) by 200 ns, collecting echoes every 400 ns. Figure 4B shows the integrated echo area normalized by the standard deviation of the noise (σ_n_). These measurements revealed that the use of deuterated buffer and glycerol led to a significant increase in the DQC echo intensity at longer t_ξ_ times, extending the duration of the signal to approximately 14 μs. Furthermore, a ca. 70% deuteration of the protein and preparation of the sample in deuterated buffer and glycerol increased the measurable DQC signal to over 20 μs. The inset of Figure 4B shows the DQC echoes for each sample at a dipolar evolution time of 5.2 μs, highlighting a substantial increase in echo intensity when using deuterated buffer, with an even greater enhancement in the deuterated protein sample. Thus, deuteration can lead to large gains in sensitivity, particularly in long-distance measurements, where the maximum reliable distance scales with the cubic root of the maximum dipolar evolution time.^109,110^ These results are not surprising given earlier work with DEER.^20,104,105^

We fit the data in Figure 4B to an expression that incorporates contributions from both phase memory time and instantaneous diffusion. Using recent theory, the decay of the DQC signal follows the exponential relationship,^63^

1 $$\mathrm{DQC echo intensity}\propto \exp\left(-{\left(\frac{2t_{m}}{T_{M}}\right)}^{\beta }\right)\times \exp\left(-4C\eta t_{m}\right)$$

where t_m_ is a pulse separation in the 6-pulse DQC sequence, T_M_ is the phase memory time, β is the stretch parameter, C is the effective doubly spin-labeled protein concentration, and η is the constant 4π^2^ω_0_/9√3. In Expression 1, the first term is due to the phase memory time, and the second term accounts for instantaneous diffusion. The predicted echo intensities were calculated using the measured T_M_ values of 3.3, 5.5, and 7.0 μs with increasing deuteration levels (Figure S5). To achieve an optimal fit to the experimental data, the parameter C was adjusted in the instantaneous diffusion term, with all three data sets fitting well using an effective concentration of 20 μM. The decrease in DQC signal with the dipolar evolution time is in good agreement with theoretical predictions.

The extended T_M_ in deuterated nitroxide samples provides a major advantage over nondeuterated samples, where T_M_ typically ranges from 2 to 4 μs,^104^ limiting measurable distances and sensitivity.^111^ Note that the enhanced relaxation times in this study are not representative given the incomplete extent of deuteration. In addition, the increase in T_M_ can depend on a number of factors, including the extent of deuteration and proximity of the spin label to the protein surface and solvent matrix. The use of deuterated buffer and cryoprotectant in nitroxide-labeled protein solutions has been shown to increase the T_M_ to around 5–6 μs,^104^ and for fully deuterated protein, values of 28–64 μs have been reported.^104,112,113^ Thus, deuteration provides an increase in sensitivity of DQC measurements and expedited acquisition times. Further, the enhanced sensitivity gained by using deuterated solution offers a way to work at lower protein concentrations.

### Combination of Optimized Parameters to Collect DQC Time Trace

Leveraging the enhanced sensitivity achieved through deuteration and optimized experimental parameters, we collected DQC time traces at the maximum of the FS-ESE for each 50 μM protein sample. Using 8 ns π pulses and carefully optimized pulse separations, we acquired the primary DQC signals in Figure 5A and the extracted distance distributions in Figure 5B. The data are shown for a 1.5 μs dipolar evolution time with a *y*-axis offset for comparison. The experimental parameters are provided in Tables S3 and S4.

**Figure 5:**
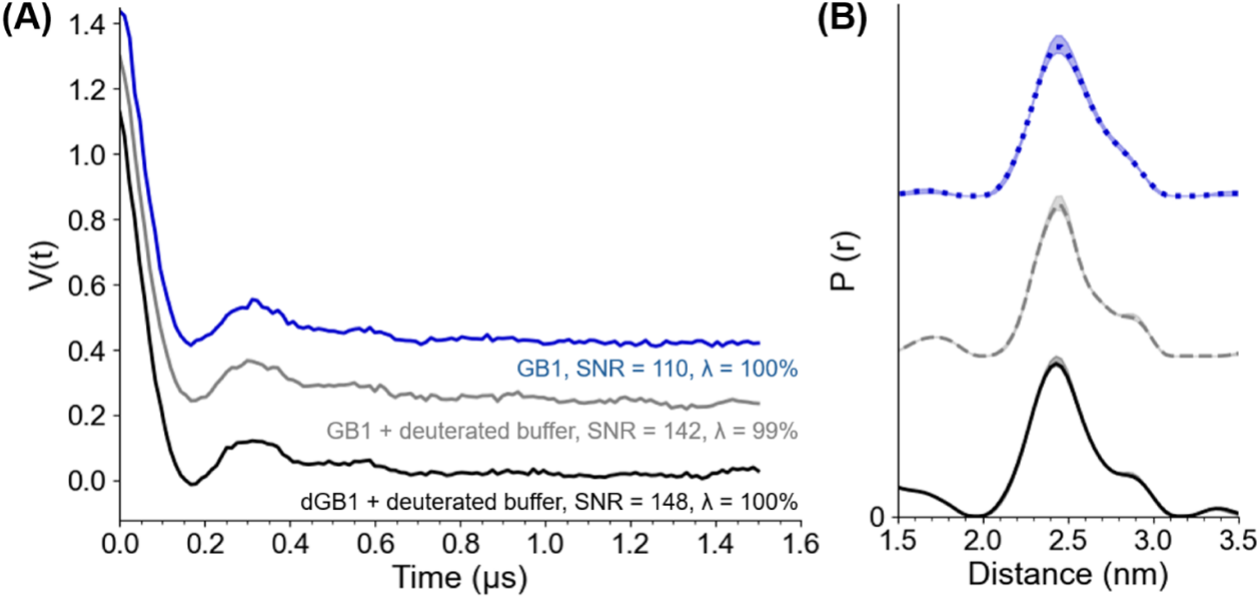
(A) DQC time traces collected at 50 K using 50 μM GB1 in nondeuterated solution (blue), deuterated buffer and glycerol (gray), and deuterated protein and solution (black) The *y*-axis is offset for ease of visualization. Each time trace was collected for 11 min with 256-step phase cycling. The SNR was calculated as the modulation depth (λ) divided by the standard deviation of the noise in the imaginary component. (B) Distance distributions obtained from the DQC signals. The *y*-axis is offset for ease of visualization. The analysis was performed using the DeerAnalysis software.^42^

We collected the time traces in Figure 5A as a single scan in only 11 min for each sample, achieving significantly high signal-to-noise ratios (SNR) of 110, 142, and 148 for the nondeuterated, deuterated buffer, and deuterated protein samples, respectively. We calculated the SNR using the equation SNR = λ/σ_rms_, where λ is the modulation depth and σ_rms_ is the standard deviation of the noise in the imaginary component after phase correction.^37,114,115^ For comparison, a minimum SNR of 20 for DEER data is accepted by the community.^37^ The distance distributions shown in Figure 5B are centered around 2.4 nm, which is consistent with previous measurements on the 15R1/28R1 GB1 sample.^116^

The high signal sensitivity in these experiments is due to multiple factors, including the large modulation depths and low background signal. As we have shown in previous work,^116^ the DQC modulation depth reached 100%, which is more than twice the modulation depths typically seen in other nitroxide-based pulsed dipolar ESR experiments like DEER and RIDME.^37,54,76^ The remarkable increase in sensitivity at Q-band was additionally made possible by the 300 W traveling-wave tube (TWT) amplifier and the large B_1_ field of the resonator. The 100% modulation depth makes DQC is an attractive technique, especially when combined with previous work that demonstrated its ability to measure distances in protein concentrations as low as 25 nM. Additionally, a comparison of the sensitivity of DQC versus DEER is provided in our previous work.^116^ The deep dipolar modulations demonstrated in this work have been shown to be particularly advantageous in resolving bimodal distance distributions of nitroxide-labeled proteins with populations as low as 12.5%.^116^ It will be interesting to study the potential effect of reduced labeling efficiency on the DQC modulation depth.

In addition to the high modulation depth, the low background component in the DQC time domain signal provides high sensitivity. Therefore, we investigated the effect of spin concentration on the DQC background signal. Pulsed ESR experiments are typically performed on nitroxide-labeled proteins at concentrations in the range of 5 to 400 μM.^76^ Figure 6 shows the DQC time traces for protein samples in deuterated buffer at 10, 50, and 100 μM, revealing only small differences in the slope of the background signal.

**Figure 6:**
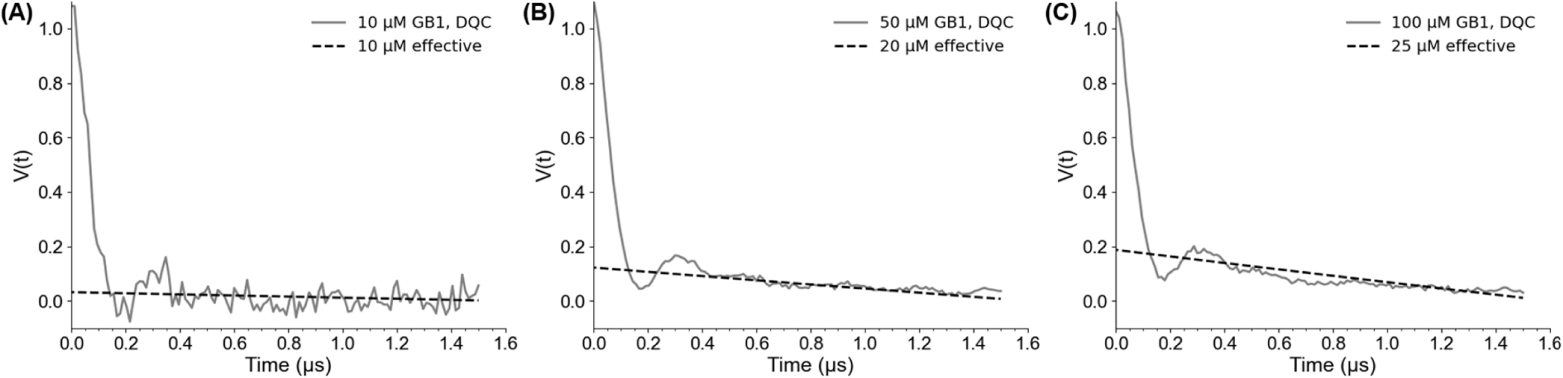
Primary DQC time traces collected using 15R1/28R1 GB1 in deuterated solution at concentrations of (A) 10, (B) 50, and (C) 100 μM. The data were fit using eq 2 with effective doubly spin-labeled protein concentrations of 10, 20, and 25 μM, respectively. The fits are shown as dashed black lines.

While high spin concentrations will increase the sensitivity of the experiment, it also increases the background decay due to intermolecular interactions. In DEER and RIDME, the background decay is separated from the intramolecular contribution by baseline correction.^37,42,58,76,117^ However, complications arise during correction, particularly the so-called noise explosion for strongly decaying signals, such as in RIDME, or oscillation dampening, which can affect analysis of longer distances.^117^ Furthermore, when extracting a distance distribution from the background-corrected signal via regularization,^42^ the solution will provide an approximation that is less accurate than the background-free solution.^117^

On the other hand, the background decay in DQC is minimal at low micromolar spin concentrations,^34,63,103^ so the signal can be estimated to be mostly due to intramolecular interactions, which reduces both user bias and errors from improper background correction. We applied a recently derived theoretical expression for the DQC background signal to our experimental results.^63^ We fit the background in the DQC signal shown in Figure 6, by varying the effective doubly spin-labeled protein concentration in the expression for the background signal:^63^

2 $$V_{BG}=\frac{C}{8}\left[\exp\left(-2C\eta \left(t_{m}+\left|t_{\xi }\right|\right)\right)-\exp\left(-4C\eta t_{m}\right)\right]$$

In eq 2, C is the effective doubly spin-labeled protein concentration, η is equal to 4π^2^ ω_0_/9 √3, *t*_*m*_ is a pulse separation from the 6-pulse DQC sequence, and *t*_ξ_ is the dipolar evolution time in the DQC signal.

The DQC signal for a 10 μM GB1 sample, as shown in Figure 6A, fits well to the background decay with an effective protein concentration of 10 μM. For the other two samples, the effective doubly labeled protein concentration was lower than the bulk concentration, as predicted by theory.^63^ Importantly, the intermolecular decay for the 50 μM sample was fit with an effective concentration, C, of 20 μM. The value is identical to that predicted from the variation of the DQC signal with dipolar evolution times (cf. Figure 4). Overall, these results show that DQC data have negligible backgrounds up to protein concentrations of 50 μM, though concentrations up to 100 μM have minimal backgrounds.

Looking ahead, it is useful to discuss the different pulsed dipolar spectroscopic methods. DEER is the most widely implemented method, and this technique benefits from a thorough understanding of potential errors in analysis and in methodology.^37–45^ However, the need for two distinct pulses can lead to compromises in the lengths of the pump and observer pulse lengths given the bandwidth of typical resonators. The use of longer pulses leads to a reduction in both modulation depth as well as sensitivity for concentration-limited samples. So far, a lower concentration limit of 100 nM has been achieved with DEER.^76,77^ The combination of orthogonal spin labeling (i.e., Cu­(II) spin labeling and trityl or nitroxide) and RIDME has enabled measurements at concentrations as low as 10 nM.^78^ In contrast, DQC may be useful to enable distance measurements at low concentrations on doubly labeled nitroxide biomolecules due to the large modulation depths and minimal background decay. Our previous work has demonstrated that these features enable reliable distance measurements at protein concentrations as low as 25 nM and allow resolution of bimodal distance distributions.^116^ The present study builds upon these findings by further elaborating Q-band nitroxide DQC.

## Conclusions

In this work, we show that Q-band DQC with doubly nitroxide-labeled proteins is a readily accessible and incisive technique. We show that eight ns π pulses are effective in the excitation of the double quantum transition, though Q-band nitroxide DQC should be achievable even when using more widely accessible 16 ns π pulses. On the other hand, measurements of orthogonally labeled proteins, especially when in combination with rigid spins labels like Cu­(II)-NTA,^86,87^ are better suited for RIDME.^78^ We show that the field dependence of the nitroxide DQC signal is rationalized by the nature of pumping the DQC and properties of the nitroxide line shape at Q-band. Furthermore, we show DQC time traces of 1.5 μs are obtained in approximately 11 min with high SNRs in the hundreds across varying levels of deuteration and concentration. It is important to note that we used a short dipolar evolution time in this work, but the addition of deuterated buffer or protein extends the phase memory time of the DQC echo, allowing for longer distance measurements. Additionally, using recent theory, we highlighted the role of instantaneous diffusion in DQC and demonstrated minimal background signal at doubly labeled protein concentrations up to 50 μM, with only a slight increase at 100 μM. Overall, these findings position nitroxide DQC as a broadly applicable and practical tool at Q-band frequencies for biophysical studies. Looking ahead, the feasibility and sensitivity of Q-band nitroxide DQC will enable biophysical explorations in increasingly complex and physiologically relevant systems.

## Supplementary Material



## Notes

Labeling efficiency, CW-ESR spectra, definitions of Euler angles, mass spectra of protein samples, calculations of deuteration efficiency, Hahn echo decays, DQC experimental parameters (PDF)

## Abbreviations

ESR: electron spin resonance
DQC: double quantum coherence
PDS: pulsed dipolar spectroscopy
GB1: B1 immunoglobulin binding domain of protein G
