# Supplementary material for: Q‑Band Double Quantum Coherence ESR for Sensitive Nitroxide-Based Distance Measurements
Source: J Phys Chem B. 2025 Jul 7;129(28):7108–18. doi: 10.1021/acs.jpcb.5c03169 (PMC12278208; doi:10.1021/acs.jpcb.5c03169)
Supplement: Supplementary file 1 [file jp5c03169_si_001.pdf]

Supporting Information

Q-band Double Quantum Coherence ESR for Sensitive Nitroxide-based Distance Measurements

*Alysia Mandato, Nicholas A. Moriglioni, and Sunil Saxena**

Department of Chemistry, University of Pittsburgh, Pittsburgh, Pennsylvania, 15260, United States

Table of Contents

Figure S1. X-band CW-ESR spectra	S3
Table S1. Labeling efficiencies of R1-labeled GB1 mutants	S3
Figure S2. DQC echo integration procedure	S3
Figure S3. Definitions of Euler angles	S4
Figure S4. Mass spectra of protein samples, calculations of deuteration efficiency	S4
Figure S5. Hahn echo decays	S5
Table S2. Hahn echo decay stretch parameters and phase memory times	S5
Table S3. General DQC acquisition parameters	S6
Table S4. Parameters for DQC experiments in Main Text, Figure 5	S6
Table S5. Parameters for DQC experiments in Main Text, Figure 6	S7
Figure S6. Impact of shot repetition time on DQC echo intensity	S7
References	S8

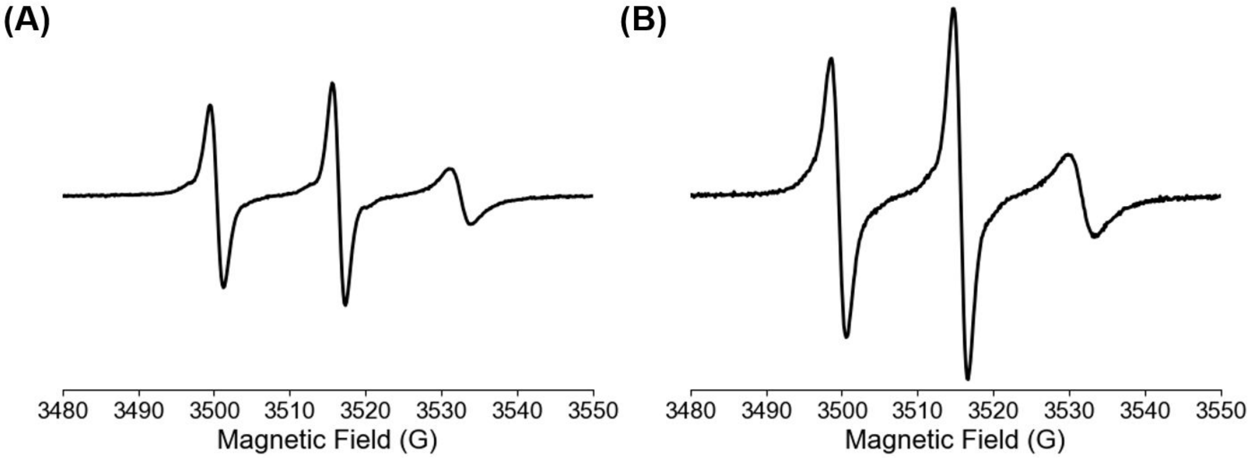

Figure S1. X-band CW-ESR of the 15R1/28R1 GB1 protein in (A) naturally abundant conditions and (B) deuterated conditions. The double integrals of the spectra were used to calculate the number of absolute spins in the Bruker XEPR software.

Table S1. Labeling efficiencies of R1-labeled GB1 samples from CW-ESR

	Protein Concentration (μM)	Spin concentration from CW-ESR (μM)	Labeling efficiency
Non-deuterated GB1	50	102	100%
Deuterated GB1	100	188	94%

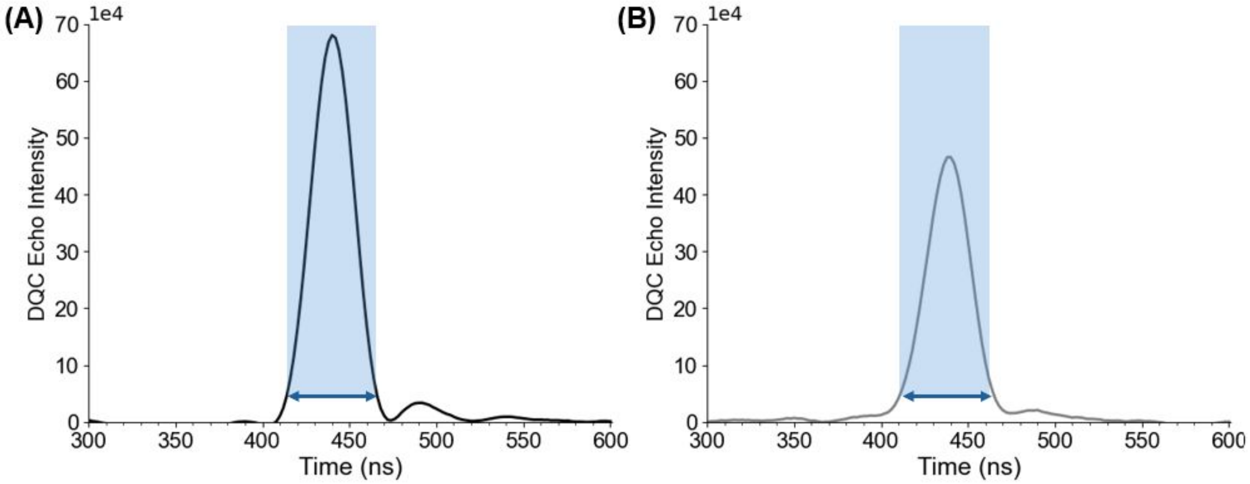

Figure S2. DQC echo integration procedure. The integration range was set based on the largest echo of the data set. From the bottom of the echo, the width of the integration range was selected slightly above the noise level of the echo, as shown by the arrow and the blue shaded region in (A). This same integration boundary was used for all other echoes in the data set, as shown in (B).

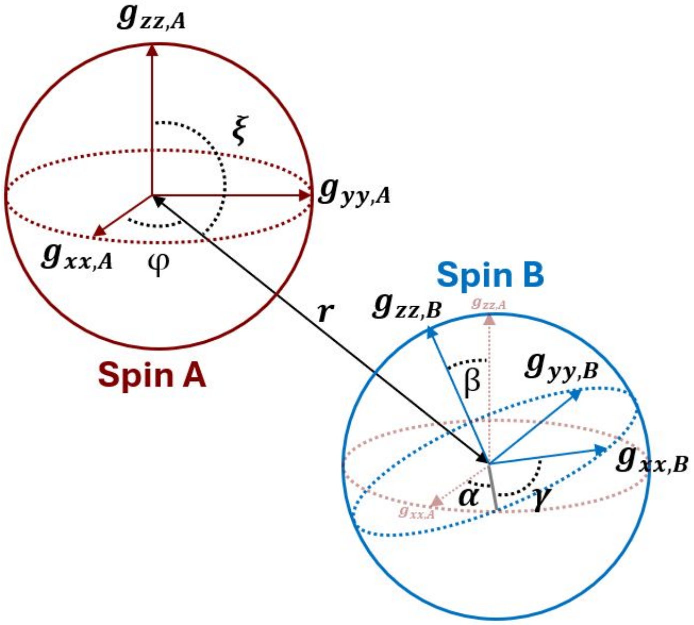

Figure S3. Representation of the relative orientations in a two-spin system. The position and orientation of the g-tensor of each spin are described by three spherical coordinates (r, ξ, φ) and three Euler angles (α, β, γ).

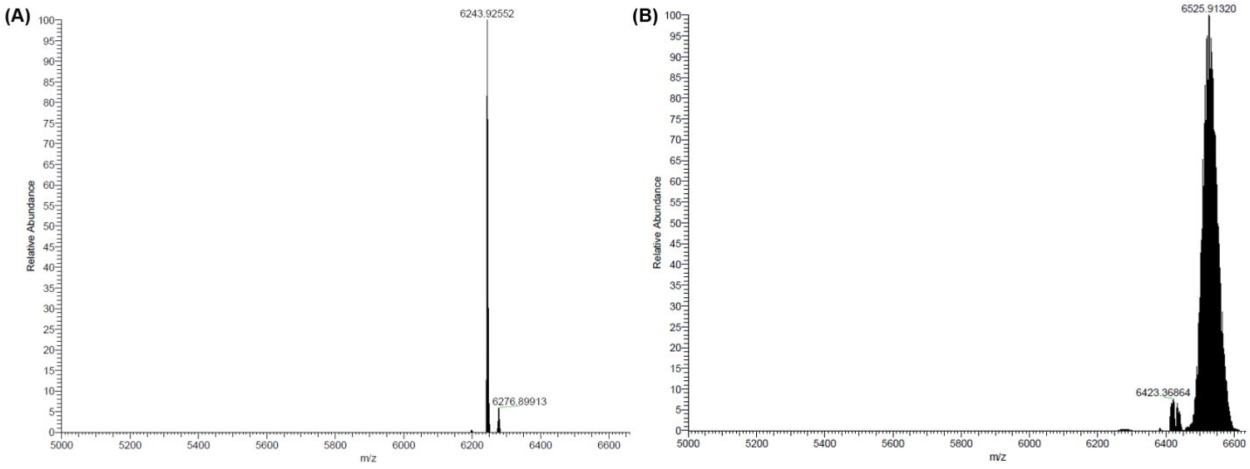

Figure S4. Mass spectra of naturally abundant 15C/28C GB1 (top) compared to deuterated 15C/28C GB1 (bottom). Mass spectra were collected using liquid chromatography-mass spectrometry and electron spray ionization. Naturally abundant GB1 has a molecular weight of 6243 g/mol. The deuterated sample has a mass centered at 6525 g/mol. This mass is consistent with an extent of deuteration of ca. 70%. The deuterated GB1 sample was stored in 1X PBS pH 7.75 in D_2O.

We determined the atomic composition of 15C/28C GB1, which contains 418 hydrogen atoms. Using the mass of naturally abundant GB1 (6243 g/mol), we substituted deuterium for hydrogen until the mass matched the dGB1 mass from the spectrum (6525 g/mol). The dGB1 mass reached

6525 g/mol when there were 294 deuterium atoms and 124 hydrogen atoms (total 418). Therefore, the extent of deuteration was calculated to be approximately 70%.

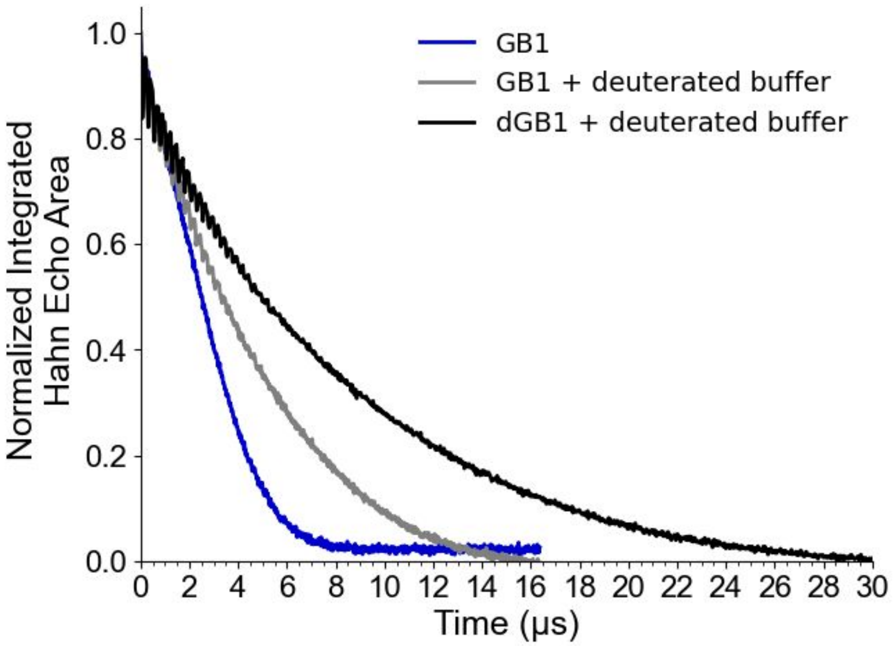

Figure S5. Hahn echo as a function of pulse separation. The pulse sequence used was $(\pi/2) - \tau - (\pi) - \tau$, and τ was increased in 8 ns increments.

Table S2. Stretch parameters for the Hahn echo decays and phase memory times of each sample.

	Stretch parameter	T_M (μs)
Non-deuterated	1.67	3.3
Deuterated buffer	1.13	5.5
Deuterated protein	0.8	7.0

Table S3. General DQC acquisition parameters

Temperature (K)	50
π/2 pulse length (ns)	4
π pulse length (ns)	8
t_p (ns)	150
t_d (ns)	106
(t_m-t_p) (ns)	1650
d30, step size (ns)	6
a, averages	13
h, shots per point	20
n, scans	1
Shot repetition time (SRT) (ms)	1
Attenuation (dB)	0
Number of points	150
Phase cycle	256-step
ESEEM modulation average*	8 steps of 16 ns

*ESEEM modulation averaging was not performed, but if deuterium ESEEM needed to be suppressed, the following protocol would be followed

Table S4. Data for DQC experiments in Main Text Figure 5

	Non-deuterated	Deuterated Buffer	Deuterated protein
GB1 concentration (μM)	50	50	50
Collection time (min)	11	11	11
Modulation depth (%)	100	99	100
Signal-to-noise ratio (SNR)†	110	142	148

†SNR = λ/σ_{rms}, where λ is the modulation depth and σ_{rms} is the standard deviation of the noise of the imaginary component after phase correction[1,2]

Table S5. Data for DQC experiments in Main Text Figure 6

	10 µM	50 µM	100 µM
Collection time (min)	11	11	11
Modulation depth (%)	100	100	100
Signal-to-noise ratio (SNR)	32	142	195

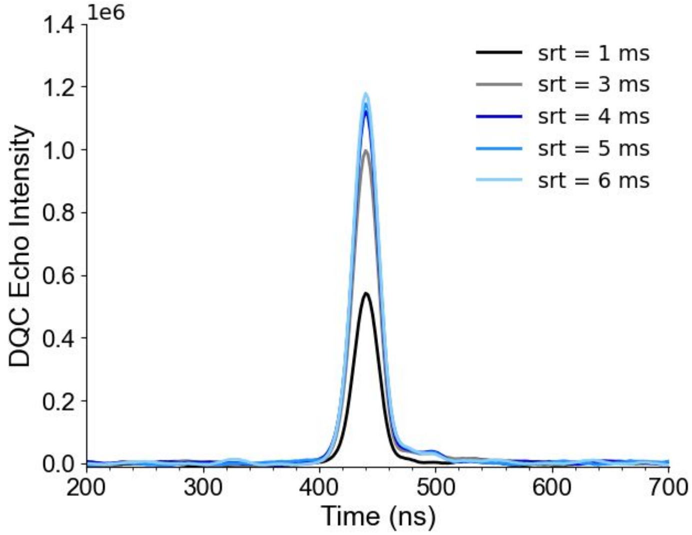

Figure S6. Impact of shot repetition time on DQC echo intensity.

The DQC echo increases by two-fold when increasing the shot repetition time from 1 ms to 3 ms. Any subsequent increases of the shot repetition time do not add much to the signal intensity while compromising run time. The ideal shot repetition time for the DQC experiment is 3 ms. However, with our current spectrometer configuration, a 3 ms shot repetition time caused errors due to lack of sufficient memory time. Therefore, experiments in this work were performed with a 1 ms srt. For nitroxide DEER measurements, the recommended “sequence repetition time” is ~5 ms at 50 K, or at least 80% of echo recovery.[2]

References

(1) Brandon, S.; Beth, A. H.; Hustedt, E. J. The Global Analysis of DEER Data. *J Magn Reson* **2012**, *218*, 93–104. https://doi.org/10.1016/j.jmr.2012.03.006.

(2) Schiemann, O.; Heubach, C. A.; Abdullin, D.; Ackermann, K.; Azarkh, M.; Bagryanskaya, E. G.; Drescher, M.; Endeward, B.; Freed, J. H.; Galazzo, L.; Goldfarb, D.; Hett, T.; Esteban Hofer, L.; Fábregas Ibáñez, L.; Hustedt, E. J.; Kucher, S.; Kuprov, I.; Lovett, J. E.; Meyer, A.; Ruthstein, S.; Saxena, S.; Stoll, S.; Timmel, C. R.; Di Valentin, M.; Mchaourab, H. S.; Prisner, T. F.; Bode, B. E.; Bordignon, E.; Bennati, M.; Jeschke, G. Benchmark Test and Guidelines for DEER/PELDOR Experiments on Nitroxide-Labeled Biomolecules. *J. Am. Chem. Soc.* **2021**, *143* (43), 17875–17890. https://doi.org/10.1021/jacs.1c07371.

(3) Sinha Roy, A.; Marohn, J. A.; Freed, J. H. An Analysis of Double-Quantum Coherence ESR in an N-Spin System: Analytical Expressions and Predictions. *J Chem Phys* **2024**, *160* (13), 134105. https://doi.org/10.1063/5.0200054.
